# Identifying geographic areas with high disease rates: when do confidence intervals for rates and a disease cluster detection method agree?

**DOI:** 10.1186/1476-072X-5-46

**Published:** 2006-10-18

**Authors:** Rhonda J Rosychuk

**Affiliations:** 1 Department of Pediatrics, University of Alberta, 9423 Aberhart Centre, Edmonton, Alberta, Canada

## Abstract

**Background:**

Geographic regions are often routinely monitored to identify areas with excess cases of disease. Further epidemiological investigations can be targeted to areas with higher disease rates than expected. Surveillance strategies typically include the calculation of sub-regional rates, and their associated confidence intervals, that are compared with the rate of the entire geographic region. More sophisticated approaches use disease cluster detection methods that require specialized software. These approaches are not the same but may lead to similar results in specific situations. A natural question arises as to when these different approaches lead to the same conclusions. We compare the Besag and Newell [1] cluster detection method, suitable for geographic areas with diverse population sizes, with confidence intervals for crude and directly standardized rates. The cluster detection method tests each area at a pre-specified cluster size. Conditions when these methods agree and disagree are provided. We use a dataset on self-inflicted injuries requiring medical attention as an illustration and give power comparisons for a variety of situations.

**Results:**

Three conditions must be satisfied for the confidence interval and cluster detection methods to both provide statistically significant higher rates for an individual administrative area. These criteria are based on observed and expected cases above specific thresholds. In our dataset, two areas are significant with both methods and one additional area is identified with the cluster detection method. Power comparisons for different scenarios suggest that the methods have similar power for detecting rates that are twice as large as the overall rate and when the overall rate and sample sizes are not too small. The cluster detection method has better power when the size of the cluster is relatively small.

**Conclusion:**

The cluster size plays a key role in the comparability of methods. The cluster detection method is preferred when the cluster size exceeds the number of cases in an administrative area or when the expected number of cases exceeds a threshold.

## Background

Health authorities typically monitor geographic areas for cases of particular diseases or conditions using population databases and disease registries. Areas with high rates can be targeted for more thorough epidemiological investigations and for health policy interventions. Two main approaches are used to determine geographic areas that have higher rates than would be expected by chance alone. The disease rate for each area can be calculated and compared with an overall rate. Confidence intervals are calculated for each rate and those areas that have confidence intervals that lie above the overall rate are considered to have high rates. Another approach is to use statistical disease cluster detection methods. Generally, these methods require specialized software to complete and additional information on the spatial relationship amongst cases. Because of the inclusion of the spatial relationship, these methods are preferred over the comparison of individual rates. Cluster detection tests are not the same as individual area disease rate comparisons, but clusters identified as a single area have a connection with the area-specific disease rate. One may ask when the cluster detection approach would provide different conclusions than the area-specific confidence interval approach. The goal of this paper is to provide conditions when the confidence interval method and a particular cluster detection method agree and disagree.

There are several different cluster detection methods available for a variety of situations (e.g., Cluster Evaluation Permutation Procedure [2], Besag and Newell test [1], spatial scan (Satscan) [3,4]; see [5] for an overview of cluster detection methods). Tests generally look at areas of similar population sizes and compare case counts or examine areas of similar case counts and compare the population providing these cases. These methods can involve non-focused or focused testing [1]. Non-focused tests identify areas with elevated cases and focused tests identify areas of excess cases near potential point sources of influence such as environmental contaminants. Of particular interest are non-focused methods that test areas with diverse population sizes [1-3,6-8]. We direct our attention to the non-focused test developed by Besag and Newell [1] because it uses area aggregate case and population data along with a simple nearest neighbour relationship. This test has the most similarities with the traditional confidence interval approach because the existing geographic boundaries are used and each area, alone or in combination with neighbours, is tested separately. Other methods, such as Kulldorff [3], are not based on tests of each area and are not comparable with the confidence interval approach.

We provide an overview of confidence interval and Besag and Newell [1] approaches that are used to identify areas of high disease rates. Conditions are shown for the agreement of approaches based on crude and directly standardized rates and demonstrated on individuals seeking medical treatment at emergency departments for self-inflicted injuries. The power of each method to detect clusters is also provided and compared.

## Methods

### Crude and directly standardized rates

Suppose that a geographical study region is comprised of *I *administrative areas, referred to as cells, and that the case and population data are stratified by *S *categories. For cell *i*, the number of population and cases in stratum *s *are denoted by *n*_*is *_and *c*_*is*_, respectively, *s *= 1,...,*S*. Summing over strata, the total number of cases and population for cell *i *are ci+=∑s=1Scis
 MathType@MTEF@5@5@+=feaafiart1ev1aaatCvAUfKttLearuWrP9MDH5MBPbIqV92AaeXatLxBI9gBaebbnrfifHhDYfgasaacH8akY=wiFfYdH8Gipec8Eeeu0xXdbba9frFj0=OqFfea0dXdd9vqai=hGuQ8kuc9pgc9s8qqaq=dirpe0xb9q8qiLsFr0=vr0=vr0dc8meaabaqaciaacaGaaeqabaqabeGadaaakeaacqWGJbWydaWgaaWcbaGaemyAaKMaey4kaScabeaakiabg2da9maaqadabaGaem4yam2aaSbaaSqaaiabdMgaPjabdohaZbqabaaabaGaem4CamNaeyypa0JaeGymaedabaGaem4uamfaniabggHiLdaaaa@3C44@ and ni+=∑s=1Snis
 MathType@MTEF@5@5@+=feaafiart1ev1aaatCvAUfKttLearuWrP9MDH5MBPbIqV92AaeXatLxBI9gBaebbnrfifHhDYfgasaacH8akY=wiFfYdH8Gipec8Eeeu0xXdbba9frFj0=OqFfea0dXdd9vqai=hGuQ8kuc9pgc9s8qqaq=dirpe0xb9q8qiLsFr0=vr0=vr0dc8meaabaqaciaacaGaaeqabaqabeGadaaakeaacqWGUbGBdaWgaaWcbaGaemyAaKMaey4kaScabeaakiabg2da9maaqadabaGaemOBa42aaSbaaSqaaiabdMgaPjabdohaZbqabaaabaGaem4CamNaeyypa0JaeGymaedabaGaem4uamfaniabggHiLdaaaa@3C70@ respectively. The total population and cases for the entire region are n++=∑i=1Ini+
 MathType@MTEF@5@5@+=feaafiart1ev1aaatCvAUfKttLearuWrP9MDH5MBPbIqV92AaeXatLxBI9gBaebbnrfifHhDYfgasaacH8akY=wiFfYdH8Gipec8Eeeu0xXdbba9frFj0=OqFfea0dXdd9vqai=hGuQ8kuc9pgc9s8qqaq=dirpe0xb9q8qiLsFr0=vr0=vr0dc8meaabaqaciaacaGaaeqabaqabeGadaaakeaacqWGUbGBdaWgaaWcbaGaey4kaSIaey4kaScabeaakiabg2da9maaqadabaGaemOBa42aaSbaaSqaaiabdMgaPjabgUcaRaqabaaabaGaemyAaKMaeyypa0JaeGymaedabaGaemysaKeaniabggHiLdaaaa@3B42@ and c++=∑i=1Ici+
 MathType@MTEF@5@5@+=feaafiart1ev1aaatCvAUfKttLearuWrP9MDH5MBPbIqV92AaeXatLxBI9gBaebbnrfifHhDYfgasaacH8akY=wiFfYdH8Gipec8Eeeu0xXdbba9frFj0=OqFfea0dXdd9vqai=hGuQ8kuc9pgc9s8qqaq=dirpe0xb9q8qiLsFr0=vr0=vr0dc8meaabaqaciaacaGaaeqabaqabeGadaaakeaacqWGJbWydaWgaaWcbaGaey4kaSIaey4kaScabeaakiabg2da9maaqadabaGaem4yam2aaSbaaSqaaiabdMgaPjabgUcaRaqabaaabaGaemyAaKMaeyypa0JaeGymaedabaGaemysaKeaniabggHiLdaaaa@3B16@, respectively, and the overall regional rate becomes *c*_++_/*n*_++ _(also referred to as overall proportion). Additionally, we calculate the number of cases and population, by strata, in the entire region, c+s=∑i=1Icis
 MathType@MTEF@5@5@+=feaafiart1ev1aaatCvAUfKttLearuWrP9MDH5MBPbIqV92AaeXatLxBI9gBaebbnrfifHhDYfgasaacH8akY=wiFfYdH8Gipec8Eeeu0xXdbba9frFj0=OqFfea0dXdd9vqai=hGuQ8kuc9pgc9s8qqaq=dirpe0xb9q8qiLsFr0=vr0=vr0dc8meaabaqaciaacaGaaeqabaqabeGadaaakeaacqWGJbWydaWgaaWcbaGaey4kaSIaem4Camhabeaakiabg2da9maaqadabaGaem4yam2aaSbaaSqaaiabdMgaPjabdohaZbqabaaabaGaemyAaKMaeyypa0JaeGymaedabaGaemysaKeaniabggHiLdaaaa@3C30@ and n+s=∑i=1Inis
 MathType@MTEF@5@5@+=feaafiart1ev1aaatCvAUfKttLearuWrP9MDH5MBPbIqV92AaeXatLxBI9gBaebbnrfifHhDYfgasaacH8akY=wiFfYdH8Gipec8Eeeu0xXdbba9frFj0=OqFfea0dXdd9vqai=hGuQ8kuc9pgc9s8qqaq=dirpe0xb9q8qiLsFr0=vr0=vr0dc8meaabaqaciaacaGaaeqabaqabeGadaaakeaacqWGUbGBdaWgaaWcbaGaey4kaSIaem4Camhabeaakiabg2da9maaqadabaGaemOBa42aaSbaaSqaaiabdMgaPjabdohaZbqabaaabaGaemyAaKMaeyypa0JaeGymaedabaGaemysaKeaniabggHiLdaaaa@3C5C@*s *= 1,...,*S*.

A crude rate is a proportion and typically, if the lower endpoint of the 95% confidence interval (CI) for the proportion is larger than the overall regional rate, then the cell is considered to have a statistically higher rate. The approximate 100(1 - *α*)% CI for the crude rate in cell *i *is

ci+ni+±z(α/2)ci+ni+2(1−ci+ni+)     (1)
 MathType@MTEF@5@5@+=feaafiart1ev1aaatCvAUfKttLearuWrP9MDH5MBPbIqV92AaeXatLxBI9gBaebbnrfifHhDYfgasaacH8akY=wiFfYdH8Gipec8Eeeu0xXdbba9frFj0=OqFfea0dXdd9vqai=hGuQ8kuc9pgc9s8qqaq=dirpe0xb9q8qiLsFr0=vr0=vr0dc8meaabaqaciaacaGaaeqabaqabeGadaaakeaadaWcaaqaaiabdogaJnaaBaaaleaacqWGPbqAcqGHRaWkaeqaaaGcbaGaemOBa42aaSbaaSqaaiabdMgaPjabgUcaRaqabaaaaOGaeyySaeRaemOEaONaeiikaGccciGae8xSdeMaei4la8IaeGOmaiJaeiykaKYaaOaaaeaadaWcaaqaaiabdogaJnaaBaaaleaacqWGPbqAcqGHRaWkaeqaaaGcbaGaemOBa42aa0baaSqaaiabdMgaPjabgUcaRaqaaiabikdaYaaaaaGcdaqadaqaaiabigdaXiabgkHiTmaalaaabaGaem4yam2aaSbaaSqaaiabdMgaPjabgUcaRaqabaaakeaacqWGUbGBdaWgaaWcbaGaemyAaKMaey4kaScabeaaaaaakiaawIcacaGLPaaaaSqabaGccaWLjaGaaCzcaiabcIcaOiabigdaXiabcMcaPaaa@54A9@

where *z*(*α*/2) is the *α*/2 quantile of the standard normal distribution. Note that for the normal approximation to be appropriate for binomial data, *n*_*i*+_*c*_++ _(*n*_++ _- *c*_++_)/n++2
 MathType@MTEF@5@5@+=feaafiart1ev1aaatCvAUfKttLearuWrP9MDH5MBPbIqV92AaeXatLxBI9gBaebbnrfifHhDYfgasaacH8akY=wiFfYdH8Gipec8Eeeu0xXdbba9frFj0=OqFfea0dXdd9vqai=hGuQ8kuc9pgc9s8qqaq=dirpe0xb9q8qiLsFr0=vr0=vr0dc8meaabaqaciaacaGaaeqabaqabeGadaaakeaacqWGUbGBdaqhaaWcbaGaey4kaSIaey4kaScabaGaeGOmaidaaaaa@30F4@ should be at least as large as 10 (e.g., [9] p. 80). For a rare disease, the corresponding cell population size would have to be relatively large.

For directly standardized rates, we need to define weights *w*_*is*_*= n*_+*s*_*/*(*n*_*is*_*n*_++_) for cell *i *and stratum *s*. The standardized rate for cell *i *becomes yi=∑s=1Swiscis
 MathType@MTEF@5@5@+=feaafiart1ev1aaatCvAUfKttLearuWrP9MDH5MBPbIqV92AaeXatLxBI9gBaebbnrfifHhDYfgasaacH8akY=wiFfYdH8Gipec8Eeeu0xXdbba9frFj0=OqFfea0dXdd9vqai=hGuQ8kuc9pgc9s8qqaq=dirpe0xb9q8qiLsFr0=vr0=vr0dc8meaabaqaciaacaGaaeqabaqabeGadaaakeaaieGacqWF5bqEdaWgaaWcbaGae8xAaKgabeaakiabg2da9maaqadabaGaem4DaC3aaSbaaSqaaiabdMgaPjabdohaZbqabaaabaGaem4CamNaeyypa0JaeGymaedabaGaem4uamfaniabggHiLdGccqWGJbWydaWgaaWcbaGaemyAaKMaem4Camhabeaaaaa@4008@ with variance vi=∑s=1Swis2cis
 MathType@MTEF@5@5@+=feaafiart1ev1aaatCvAUfKttLearuWrP9MDH5MBPbIqV92AaeXatLxBI9gBaebbnrfifHhDYfgasaacH8akY=wiFfYdH8Gipec8Eeeu0xXdbba9frFj0=OqFfea0dXdd9vqai=hGuQ8kuc9pgc9s8qqaq=dirpe0xb9q8qiLsFr0=vr0=vr0dc8meaabaqaciaacaGaaeqabaqabeGadaaakeaacqWG2bGDdaWgaaWcbaGaemyAaKgabeaakiabg2da9maaqadabaGaem4DaC3aa0baaSqaaiabdMgaPjabdohaZbqaaiabikdaYaaakiabdogaJnaaBaaaleaacqWGPbqAcqWGZbWCaeqaaaqaaiabdohaZjabg2da9iabigdaXaqaaiabdofatbqdcqGHris5aaaa@40F2@. In practice, researchers may calculate the approximate 100(1 - *α*)% CI based on the normal distribution as

*y*_*i *_± *z*(*α*/2)vi
 MathType@MTEF@5@5@+=feaafiart1ev1aaatCvAUfKttLearuWrP9MDH5MBPbIqV92AaeXatLxBI9gBaebbnrfifHhDYfgasaacH8akY=wiFfYdH8Gipec8Eeeu0xXdbba9frFj0=OqFfea0dXdd9vqai=hGuQ8kuc9pgc9s8qqaq=dirpe0xb9q8qiLsFr0=vr0=vr0dc8meaabaqaciaacaGaaeqabaqabeGadaaakeaadaGcaaqaaiabdAha2naaBaaaleaacqWGPbqAaeqaaaqabaaaaa@2FB8@.     (2)

Alternatively, Fay and Feuer [10] provide approximate 100(1 - *α*)% CIs for cell *i *based on the gamma distribution as

(vi2yixiL,vi+wiM22(yi+wiM)xiU)     (3)
 MathType@MTEF@5@5@+=feaafiart1ev1aaatCvAUfKttLearuWrP9MDH5MBPbIqV92AaeXatLxBI9gBaebbnrfifHhDYfgasaacH8akY=wiFfYdH8Gipec8Eeeu0xXdbba9frFj0=OqFfea0dXdd9vqai=hGuQ8kuc9pgc9s8qqaq=dirpe0xb9q8qiLsFr0=vr0=vr0dc8meaabaqaciaacaGaaeqabaqabeGadaaakeaadaqadaqaamaalaaabaGaemODay3aaSbaaSqaaiabdMgaPbqabaaakeaacqaIYaGmcqWG5bqEdaWgaaWcbaGaemyAaKgabeaaaaGccqWG4baEdaWgaaWcbaGaemyAaKMaemitaWeabeaakiabcYcaSmaalaaabaGaemODay3aaSbaaSqaaiabdMgaPbqabaGccqGHRaWkcqWG3bWDdaqhaaWcbaGaemyAaKMaemyta0eabaGaeGOmaidaaaGcbaGaeGOmaiJaeiikaGIaemyEaK3aaSbaaSqaaiabdMgaPbqabaGccqGHRaWkcqWG3bWDdaWgaaWcbaGaemyAaKMaemyta0eabeaakiabcMcaPaaacqWG4baEdaWgaaWcbaGaemyAaKMaemyvaufabeaaaOGaayjkaiaawMcaaiaaxMaacaWLjaGaeiikaGIaeG4mamJaeiykaKcaaa@564E@

where *w*_*iM *_= max_*s*∈{1,...,*S*}_(*w*_*is*_), *x*_*iL *_is the *α*/2 quantile of the χ^2 ^distribution with 2yi2
 MathType@MTEF@5@5@+=feaafiart1ev1aaatCvAUfKttLearuWrP9MDH5MBPbIqV92AaeXatLxBI9gBaebbnrfifHhDYfgasaacH8akY=wiFfYdH8Gipec8Eeeu0xXdbba9frFj0=OqFfea0dXdd9vqai=hGuQ8kuc9pgc9s8qqaq=dirpe0xb9q8qiLsFr0=vr0=vr0dc8meaabaqaciaacaGaaeqabaqabeGadaaakeaacqaIYaGmcqWG5bqEdaqhaaWcbaGaemyAaKgabaGaeGOmaidaaaaa@3193@/*v*_*i *_degrees of freedom, and *x*_*iU *_is the 1 - *α*/2 quantile of the χ^2 ^distribution with 2(*y*_*i *_+ *w*_*iM*_)^2^/(*v*_*i *_+ *w*_*iM*_) degrees of freedom. Regardless of the method, if the overall regional rate is smaller than the lower limit of the CI, the cell is considered to have a statistically higher rate. We refer to these CI approaches as the CI method and Patel et al. [11] have included this type of analysis in their examination of septo-optic dysplasia and optic nerve hypoplasia.

### Besag and Newell method

In addition to the population and case counts, the Besag and Newell (BN) method requires a rough spatial relationship among the cells based on pairwise distances between cell centroids. For cell *i*, the remaining cells are ordered according to increased distance from the cell *i *centroid. Let cell *i*_*p *_be the *p*-th closest cell to cell *i*, *p *∈ {1,..., *I *- 1}, and define *i*_0 _= *i*.

A cluster size is pre-specified and each cell is tested separately. Suppose that the cluster size for cell *i *is *k*_*i*_. Note that the *k*_*i *_need not be unique and the situation may be that *k*_*i *_= *k *for all *i*. For this test, the null hypothesis is that every individual is equally likely to be a case independent of other individuals and the location of residence. The observed test statistic (ℓ) for cell *i *is the number of cells that must be combined with cell *i*, to include the nearest *k*_*i *_cases,

min⁡{q suchthat ki≤∑p=0qcip+}.     (4)
 MathType@MTEF@5@5@+=feaafiart1ev1aaatCvAUfKttLearuWrP9MDH5MBPbIqV92AaeXatLxBI9gBaebbnrfifHhDYfgasaacH8akY=wiFfYdH8Gipec8Eeeu0xXdbba9frFj0=OqFfea0dXdd9vqai=hGuQ8kuc9pgc9s8qqaq=dirpe0xb9q8qiLsFr0=vr0=vr0dc8meaabaqaciaacaGaaeqabaqabeGadaaakeaacyGGTbqBcqGGPbqAcqGGUbGBdaGadeqaaiabdghaXjabbccaGGqaaiab=nhaZjab=vha1jab=ngaJjab=HgaOjab=bcaGiab=rha0jab=HgaOjab=fgaHjab=rha0jabbccaGiabdUgaRnaaBaaaleaacqWGPbqAaeqaaOGaeyizIm6aaabCaeaacqWGJbWydaWgaaWcbaGaemyAaK2aaSbaaWqaaiabdchaWbqabaWccqGHRaWkaeqaaaqaaiabdchaWjabg2da9iabicdaWaqaaiabdghaXbqdcqGHris5aaGccaGL7bGaayzFaaGaeiOla4IaaCzcaiaaxMaacqGGOaakcqaI0aancqGGPaqkaaa@578E@

The basic form of the probabilities for the significance level are the same when strata are ignored or included in the analysis. The number of cases in cell *i *and its nearest ℓ neighbours is approximated by a Poisson distribution and the significance level is

1−∑u=0ki−1λi:ℓuu!e−λi:ℓ.     (5)
 MathType@MTEF@5@5@+=feaafiart1ev1aaatCvAUfKttLearuWrP9MDH5MBPbIqV92AaeXatLxBI9gBaebbnrfifHhDYfgasaacH8akY=wiFfYdH8Gipec8Eeeu0xXdbba9frFj0=OqFfea0dXdd9vqai=hGuQ8kuc9pgc9s8qqaq=dirpe0xb9q8qiLsFr0=vr0=vr0dc8meaabaqaciaacaGaaeqabaqabeGadaaakeaacqaIXaqmcqGHsisldaaeWbqaamaalaaabaacciGae83UdW2aa0baaSqaaiabdMgaPjabcQda6iabloriSbqaaiabdwha1baaaOqaaiabdwha1jabcgcaHaaaaSqaaiabdwha1jabg2da9iabicdaWaqaaiabdUgaRnaaBaaameaacqWGPbqAaeqaaSGaeyOeI0IaeGymaedaniabggHiLdGccqWGLbqzdaahaaWcbeqaaiabgkHiTiab=T7aSnaaBaaameaacqWGPbqAcqGG6aGocqWItecBaeqaaaaakiabc6caUiaaxMaacaWLjaWaaeWaaeaacqaI1aqnaiaawIcacaGLPaaaaaa@4EC9@

When stratification is ignored, *λ*_*i*:ℓ _is estimated by λ^i:ℓ=∑p=0ℓnip+c++/n++
 MathType@MTEF@5@5@+=feaafiart1ev1aaatCvAUfKttLearuWrP9MDH5MBPbIqV92AaeXatLxBI9gBaebbnrfifHhDYfgasaacH8akY=wiFfYdH8Gipec8Eeeu0xXdbba9frFj0=OqFfea0dXdd9vqai=hGuQ8kuc9pgc9s8qqaq=dirpe0xb9q8qiLsFr0=vr0=vr0dc8meaabaqaciaacaGaaeqabaqabeGadaaakeaacuaH7oaBgaqcamaaBaaaleaacqWGPbqAcqGG6aGocqWItecBaeqaaOGaeyypa0ZaaabmaeaacqWGUbGBdaWgaaWcbaGaemyAaK2aaSbaaWqaaiabdchaWbqabaWccqGHRaWkaeqaaOGaem4yam2aaSbaaSqaaiabgUcaRiabgUcaRaqabaGccqGGVaWlcqWGUbGBdaWgaaWcbaGaey4kaSIaey4kaScabeaaaeaacqWGWbaCcqGH9aqpcqaIWaamaeaacqWItecBa0GaeyyeIuoaaaa@46B6@. With strata, the significance level has the same form as in (5) except that *λ*_*i*:ℓ _is replaced by λ^i:ℓ∗=∑s=1S∑p=0ℓnipsc+s/n+s
 MathType@MTEF@5@5@+=feaafiart1ev1aaatCvAUfKttLearuWrP9MDH5MBPbIqV92AaeXatLxBI9gBaebbnrfifHhDYfgasaacH8akY=wiFfYdH8Gipec8Eeeu0xXdbba9frFj0=OqFfea0dXdd9vqai=hGuQ8kuc9pgc9s8qqaq=dirpe0xb9q8qiLsFr0=vr0=vr0dc8meaabaqaciaacaGaaeqabaqabeGadaaakeaaiiGacuWF7oaBgaqcamaaDaaaleaacqWGPbqAcqGG6aGocqWItecBaeaacqGHxiIkaaGccqGH9aqpdaaeWaqaamaaqadabaGaemOBa42aaSbaaSqaaiabdMgaPnaaBaaameaacqWGWbaCaeqaaSGaem4CamhabeaakiabdogaJnaaBaaaleaacqGHRaWkcqWGZbWCaeqaaOGaei4la8IaemOBa42aaSbaaSqaaiabgUcaRiabdohaZbqabaaabaGaemiCaaNaeyypa0JaeGimaadabaGaeS4eHWganiabggHiLdaaleaacqWGZbWCcqGH9aqpcqaIXaqmaeaacqWGtbWua0GaeyyeIuoaaaa@4FEA@ to account for the stratification. If the significance level is less than *α*, cell *i *and its ℓ nearest neighbours are considered to have higher rates than could be expected by chance alone and are identified as clusters.

As with the CI method, an approximation to binomial data is used. The BN method uses a Poisson approximation, appropriate in situations where the overall proportion is small and the cell population size is large (e.g., [9] p. 33). Unlike the CI method, the BN method does not require the individual cell population sizes to be relative large since it combines neighbouring cells to achieve a certain number of cases. In addition, the BN method allows for neighbouring cells to be combined and tested rather than restricting the test to an individual cell as in the CI method. The BN method has been used in a variety of clustering investigations including the geographic distribution of variant Creutzfeldt-Jakob disease in the UK [12].

### Agreement of CI and BN methods

The previous sections described two ways that cells could be identified as having statistically higher rates. If a cell is identified as having a high rate by both methods, we say that the approaches agree. We next provide the conditions when the methods will disagree and agree.

The BN method involves the combination of cells. If cell *i *needs to be added with one or more neighbours to contain at least *k*_*i *_cases, then ℓ > 0 and the methods are not directly comparable. That is, the CI method would not be based on a combination of cells and thus, the approaches are testing different aspects and would not both identify the same cell as having a statistically higher rate. It is a strength of the BN method that geographic areas are combined and tested. The combination allows the BN method to be less restrictive on the boundaries of a cluster, which is particularly important when the cells have small population sizes and not enough cases to be considered clusters on their own. Thus, the only way that the methods can agree is if cell combination is not required, ℓ = 0. We restrict our attention to that situation.

Consider the situation without strata. Suppose the test statistic for cell *i *is ℓ = 0 for the BN method. This test statistic implies that *k*_*i *_≤ *c*_*i*+_. For cell *i *to be identified as a cluster at significance level *α*, the significance level from (5) must be less than *α*,

1−∑u=0ki−1λ^i:0uu!e−λ^i:0<α.     (6)
 MathType@MTEF@5@5@+=feaafiart1ev1aaatCvAUfKttLearuWrP9MDH5MBPbIqV92AaeXatLxBI9gBaebbnrfifHhDYfgasaacH8akY=wiFfYdH8Gipec8Eeeu0xXdbba9frFj0=OqFfea0dXdd9vqai=hGuQ8kuc9pgc9s8qqaq=dirpe0xb9q8qiLsFr0=vr0=vr0dc8meaabaqaciaacaGaaeqabaqabeGadaaakeaacqaIXaqmcqGHsisldaaeWbqaamaalaaabaacciGaf83UdWMbaKaadaqhaaWcbaGaemyAaKMaeiOoaOJaeGimaadabaGaemyDauhaaaGcbaGaemyDauNaeiyiaecaaaWcbaGaemyDauNaeyypa0JaeGimaadabaGaem4AaS2aaSbaaWqaaiabdMgaPbqabaWccqGHsislcqaIXaqma0GaeyyeIuoakiabdwgaLnaaCaaaleqabaGaeyOeI0Iaf83UdWMbaKaadaWgaaadbaGaemyAaKMaeiOoaOJaeGimaadabeaaaaGccqGH8aapcqWFXoqycqGGUaGlcaWLjaGaaCzcamaabmaabaGaeGOnaydacaGLOaGaayzkaaaaaa@5103@

Since *k*_*i *_≤ *c*_*i*+_, we also have that

1−∑u=0ci+−1λ^i:0uu!e−λ^i:0≤1−∑u=0ki−1λ^i:0uu!e−λ^i:0<α.     (7)
 MathType@MTEF@5@5@+=feaafiart1ev1aaatCvAUfKttLearuWrP9MDH5MBPbIqV92AaeXatLxBI9gBaebbnrfifHhDYfgasaacH8akY=wiFfYdH8Gipec8Eeeu0xXdbba9frFj0=OqFfea0dXdd9vqai=hGuQ8kuc9pgc9s8qqaq=dirpe0xb9q8qiLsFr0=vr0=vr0dc8meaabaqaciaacaGaaeqabaqabeGadaaakeaacqaIXaqmcqGHsisldaaeWbqaamaalaaabaacciGaf83UdWMbaKaadaqhaaWcbaGaemyAaKMaeiOoaOJaeGimaadabaGaemyDauhaaaGcbaGaemyDauNaeiyiaecaaaWcbaGaemyDauNaeyypa0JaeGimaadabaGaem4yam2aaSbaaWqaaiabdMgaPjabgUcaRaqabaWccqGHsislcqaIXaqma0GaeyyeIuoakiabdwgaLnaaCaaaleqabaGaeyOeI0Iaf83UdWMbaKaadaWgaaadbaGaemyAaKMaeiOoaOJaeGimaadabeaaaaGccqGHKjYOcqaIXaqmcqGHsisldaaeWbqaamaalaaabaGaf83UdWMbaKaadaqhaaWcbaGaemyAaKMaeiOoaOJaeGimaadabaGaemyDauhaaaGcbaGaemyDauNaeiyiaecaaaWcbaGaemyDauNaeyypa0JaeGimaadabaGaem4AaS2aaSbaaWqaaiabdMgaPbqabaWccqGHsislcqaIXaqma0GaeyyeIuoakiabdwgaLnaaCaaaleqabaGaeyOeI0Iaf83UdWMbaKaadaWgaaadbaGaemyAaKMaeiOoaOJaeGimaadabeaaaaGccqGH8aapcqWFXoqycqGGUaGlcaWLjaGaaCzcamaabmaabaGaeG4naCdacaGLOaGaayzkaaaaaa@708E@

Hence, for cell *i *to be identified as a cluster using the BN method, *c*_*i*+ _- 1 and *k*_*i *_- 1 must both be at least as large as P
 MathType@MTEF@5@5@+=feaafiart1ev1aaatCvAUfKttLearuWrP9MDH5MBPbIqV92AaeXatLxBI9gBamrtHrhAL1wy0L2yHvtyaeHbnfgDOvwBHrxAJfwnaebbnrfifHhDYfgasaacH8akY=wiFfYdH8Gipec8Eeeu0xXdbba9frFj0=OqFfea0dXdd9vqai=hGuQ8kuc9pgc9s8qqaq=dirpe0xb9q8qiLsFr0=vr0=vr0dc8meaabaqaciaacaGaaeqabaWaaeGaeaaakeGabaaUrGqaciab=bfaqbaa@385B@_*α*_(λ^
 MathType@MTEF@5@5@+=feaafiart1ev1aaatCvAUfKttLearuWrP9MDH5MBPbIqV92AaeXatLxBI9gBaebbnrfifHhDYfgasaacH8akY=wiFfYdH8Gipec8Eeeu0xXdbba9frFj0=OqFfea0dXdd9vqai=hGuQ8kuc9pgc9s8qqaq=dirpe0xb9q8qiLsFr0=vr0=vr0dc8meaabaqaciaacaGaaeqabaqabeGadaaakeaaiiGacuWF7oaBgaqcaaaa@2E77@_*i*:0_), where P
 MathType@MTEF@5@5@+=feaafiart1ev1aaatCvAUfKttLearuWrP9MDH5MBPbIqV92AaeXatLxBI9gBamrtHrhAL1wy0L2yHvtyaeHbnfgDOvwBHrxAJfwnaebbnrfifHhDYfgasaacH8akY=wiFfYdH8Gipec8Eeeu0xXdbba9frFj0=OqFfea0dXdd9vqai=hGuQ8kuc9pgc9s8qqaq=dirpe0xb9q8qiLsFr0=vr0=vr0dc8meaabaqaciaacaGaaeqabaWaaeGaeaaakeGabaaUrGqaciab=bfaqbaa@385B@_*α*_(λ^
 MathType@MTEF@5@5@+=feaafiart1ev1aaatCvAUfKttLearuWrP9MDH5MBPbIqV92AaeXatLxBI9gBaebbnrfifHhDYfgasaacH8akY=wiFfYdH8Gipec8Eeeu0xXdbba9frFj0=OqFfea0dXdd9vqai=hGuQ8kuc9pgc9s8qqaq=dirpe0xb9q8qiLsFr0=vr0=vr0dc8meaabaqaciaacaGaaeqabaqabeGadaaakeaaiiGacuWF7oaBgaqcaaaa@2E77@_*i*:0_) is defined as the 100(1 - *α*) percentile of the Poisson distribution with estimated mean λ^
 MathType@MTEF@5@5@+=feaafiart1ev1aaatCvAUfKttLearuWrP9MDH5MBPbIqV92AaeXatLxBI9gBaebbnrfifHhDYfgasaacH8akY=wiFfYdH8Gipec8Eeeu0xXdbba9frFj0=OqFfea0dXdd9vqai=hGuQ8kuc9pgc9s8qqaq=dirpe0xb9q8qiLsFr0=vr0=vr0dc8meaabaqaciaacaGaaeqabaqabeGadaaakeaaiiGacuWF7oaBgaqcaaaa@2E77@_*i*:0 _= *n*_*i*+_*c*_++_/*n*_++_.

Cell *i *is considered to have a high rate, if the approximate 100(1 - *α*)% CI is above the overall regional rate, c_++_/*n*_++_. Using (1) we have that,

c++n++<ci+ni+−z(α/2)ci+ni+2(1−ci+ni+).     (8)
 MathType@MTEF@5@5@+=feaafiart1ev1aaatCvAUfKttLearuWrP9MDH5MBPbIqV92AaeXatLxBI9gBaebbnrfifHhDYfgasaacH8akY=wiFfYdH8Gipec8Eeeu0xXdbba9frFj0=OqFfea0dXdd9vqai=hGuQ8kuc9pgc9s8qqaq=dirpe0xb9q8qiLsFr0=vr0=vr0dc8meaabaqaciaacaGaaeqabaqabeGadaaakeaadaWcaaqaaiabdogaJnaaBaaaleaacqGHRaWkcqGHRaWkaeqaaaGcbaGaemOBa42aaSbaaSqaaiabgUcaRiabgUcaRaqabaaaaOGaeyipaWZaaSaaaeaacqWGJbWydaWgaaWcbaGaemyAaKMaey4kaScabeaaaOqaaiabd6gaUnaaBaaaleaacqWGPbqAcqGHRaWkaeqaaaaakiabgkHiTiabdQha6jabcIcaOGGaciab=f7aHjabc+caViabikdaYiabcMcaPmaakaaabaWaaSaaaeaacqWGJbWydaWgaaWcbaGaemyAaKMaey4kaScabeaaaOqaaiabd6gaUnaaDaaaleaacqWGPbqAcqGHRaWkaeaacqaIYaGmaaaaaOWaaeWaaeaacqaIXaqmcqGHsisldaWcaaqaaiabdogaJnaaBaaaleaacqWGPbqAcqGHRaWkaeqaaaGcbaGaemOBa42aaSbaaSqaaiabdMgaPjabgUcaRaqabaaaaaGccaGLOaGaayzkaaaaleqaaOGaeiOla4IaaCzcaiaaxMaadaqadaqaaiabiIda4aGaayjkaiaawMcaaaaa@5C2D@

Multiplying by *n*_*i*+_, we get

λ^i:0=ni+c++n++<ci+−z(α/2)ci+(ni+−ci+)ni+     (9)
 MathType@MTEF@5@5@+=feaafiart1ev1aaatCvAUfKttLearuWrP9MDH5MBPbIqV92AaeXatLxBI9gBaebbnrfifHhDYfgasaacH8akY=wiFfYdH8Gipec8Eeeu0xXdbba9frFj0=OqFfea0dXdd9vqai=hGuQ8kuc9pgc9s8qqaq=dirpe0xb9q8qiLsFr0=vr0=vr0dc8meaabaqaciaacaGaaeqabaqabeGadaaakeaaiiGacuWF7oaBgaqcamaaBaaaleaacqWGPbqAcqGG6aGocqaIWaamaeqaaOGaeyypa0JaemOBa42aaSbaaSqaaiabdMgaPjabgUcaRaqabaGcdaWcaaqaaiabdogaJnaaBaaaleaacqGHRaWkcqGHRaWkaeqaaaGcbaGaemOBa42aaSbaaSqaaiabgUcaRiabgUcaRaqabaaaaOGaeyipaWJaem4yam2aaSbaaSqaaiabdMgaPjabgUcaRaqabaGccqGHsislcqWG6bGEcqGGOaakcqWFXoqycqGGVaWlcqaIYaGmcqGGPaqkdaGcaaqaamaalaaabaGaem4yam2aaSbaaSqaaiabdMgaPjabgUcaRaqabaGccqGGOaakcqWGUbGBdaWgaaWcbaGaemyAaKMaey4kaScabeaakiabgkHiTiabdogaJnaaBaaaleaacqWGPbqAcqGHRaWkaeqaaOGaeiykaKcabaGaemOBa42aaSbaaSqaaiabdMgaPjabgUcaRaqabaaaaaqabaGccaWLjaGaaCzcamaabmaabaGaeGyoaKdacaGLOaGaayzkaaaaaa@5F9C@

as an additional requirement for significance of cell *i*. Thus, for the CI and BN methods to agree that cell *i *has a statistically higher rate than the overall regional rate, the following conditions must all be satisfied:

R1: *k*_*i *_≤ *c*_*i*+_

R2: P
 MathType@MTEF@5@5@+=feaafiart1ev1aaatCvAUfKttLearuWrP9MDH5MBPbIqV92AaeXatLxBI9gBamrtHrhAL1wy0L2yHvtyaeHbnfgDOvwBHrxAJfwnaebbnrfifHhDYfgasaacH8akY=wiFfYdH8Gipec8Eeeu0xXdbba9frFj0=OqFfea0dXdd9vqai=hGuQ8kuc9pgc9s8qqaq=dirpe0xb9q8qiLsFr0=vr0=vr0dc8meaabaqaciaacaGaaeqabaWaaeGaeaaakeGabaaUrGqaciab=bfaqbaa@385B@_*α*_(λ^
 MathType@MTEF@5@5@+=feaafiart1ev1aaatCvAUfKttLearuWrP9MDH5MBPbIqV92AaeXatLxBI9gBaebbnrfifHhDYfgasaacH8akY=wiFfYdH8Gipec8Eeeu0xXdbba9frFj0=OqFfea0dXdd9vqai=hGuQ8kuc9pgc9s8qqaq=dirpe0xb9q8qiLsFr0=vr0=vr0dc8meaabaqaciaacaGaaeqabaqabeGadaaakeaaiiGacuWF7oaBgaqcaaaa@2E77@_*i*:0_) ≤ *c*_*i*+ _- 1, where is the P
 MathType@MTEF@5@5@+=feaafiart1ev1aaatCvAUfKttLearuWrP9MDH5MBPbIqV92AaeXatLxBI9gBamrtHrhAL1wy0L2yHvtyaeHbnfgDOvwBHrxAJfwnaebbnrfifHhDYfgasaacH8akY=wiFfYdH8Gipec8Eeeu0xXdbba9frFj0=OqFfea0dXdd9vqai=hGuQ8kuc9pgc9s8qqaq=dirpe0xb9q8qiLsFr0=vr0=vr0dc8meaabaqaciaacaGaaeqabaWaaeGaeaaakeGabaaUrGqaciab=bfaqbaa@385B@_*α*_(λ^
 MathType@MTEF@5@5@+=feaafiart1ev1aaatCvAUfKttLearuWrP9MDH5MBPbIqV92AaeXatLxBI9gBaebbnrfifHhDYfgasaacH8akY=wiFfYdH8Gipec8Eeeu0xXdbba9frFj0=OqFfea0dXdd9vqai=hGuQ8kuc9pgc9s8qqaq=dirpe0xb9q8qiLsFr0=vr0=vr0dc8meaabaqaciaacaGaaeqabaqabeGadaaakeaaiiGacuWF7oaBgaqcaaaa@2E77@_*i*:0_) is the 100(1 - *α*) percentile of the Poisson distribution with estimated mean λ^
 MathType@MTEF@5@5@+=feaafiart1ev1aaatCvAUfKttLearuWrP9MDH5MBPbIqV92AaeXatLxBI9gBaebbnrfifHhDYfgasaacH8akY=wiFfYdH8Gipec8Eeeu0xXdbba9frFj0=OqFfea0dXdd9vqai=hGuQ8kuc9pgc9s8qqaq=dirpe0xb9q8qiLsFr0=vr0=vr0dc8meaabaqaciaacaGaaeqabaqabeGadaaakeaaiiGacuWF7oaBgaqcaaaa@2E77@_*i*:0 _= *n*_*i*+ _*c*_++_/*n*_++_

R3: λ^
 MathType@MTEF@5@5@+=feaafiart1ev1aaatCvAUfKttLearuWrP9MDH5MBPbIqV92AaeXatLxBI9gBaebbnrfifHhDYfgasaacH8akY=wiFfYdH8Gipec8Eeeu0xXdbba9frFj0=OqFfea0dXdd9vqai=hGuQ8kuc9pgc9s8qqaq=dirpe0xb9q8qiLsFr0=vr0=vr0dc8meaabaqaciaacaGaaeqabaqabeGadaaakeaaiiGacuWF7oaBgaqcaaaa@2E77@_*i*:0 _<*c*_*i*+ _- *z*(*α*/2)ci+(ni+−ci+)/ni+
 MathType@MTEF@5@5@+=feaafiart1ev1aaatCvAUfKttLearuWrP9MDH5MBPbIqV92AaeXatLxBI9gBaebbnrfifHhDYfgasaacH8akY=wiFfYdH8Gipec8Eeeu0xXdbba9frFj0=OqFfea0dXdd9vqai=hGuQ8kuc9pgc9s8qqaq=dirpe0xb9q8qiLsFr0=vr0=vr0dc8meaabaqaciaacaGaaeqabaqabeGadaaakeaadaGcaaqaaiabdogaJnaaBaaaleaacqWGPbqAcqGHRaWkaeqaaOGaeiikaGIaemOBa42aaSbaaSqaaiabdMgaPjabgUcaRaqabaGccqGHsislcqWGJbWydaWgaaWcbaGaemyAaKMaey4kaScabeaakiabcMcaPiabc+caViabd6gaUnaaBaaaleaacqWGPbqAcqGHRaWkaeqaaaqabaaaaa@3F6B@

Note that if R3 is satisfied, the tested cell will have a significantly higher rate under the CI method.

The criteria are easily adapted for the situation with *S *strata. Again, assume that the test statistic for cell *i *is ℓ = 0 for the BN method, which still implies *k*_*i *_≤ *c*_*i*+_. Cell *i *is identified as a cluster if *c*_*i*+ _- 1 is at least as large as the 100(1 - *α*) percentile of the Poisson distribution with mean λ^i:0∗=∑s=1Snisc+s/n+s
 MathType@MTEF@5@5@+=feaafiart1ev1aaatCvAUfKttLearuWrP9MDH5MBPbIqV92AaeXatLxBI9gBaebbnrfifHhDYfgasaacH8akY=wiFfYdH8Gipec8Eeeu0xXdbba9frFj0=OqFfea0dXdd9vqai=hGuQ8kuc9pgc9s8qqaq=dirpe0xb9q8qiLsFr0=vr0=vr0dc8meaabaqaciaacaGaaeqabaqabeGadaaakeaaiiGacuWF7oaBgaqcamaaDaaaleaacqWGPbqAcqGG6aGocqaIWaamaeaacqGHxiIkaaGccqGH9aqpdaaeWaqaaiabd6gaUnaaBaaaleaacqWGPbqAcqWGZbWCaeqaaOGaem4yam2aaSbaaSqaaiabgUcaRiabdohaZbqabaaabaGaem4CamNaeyypa0JaeGymaedabaGaem4uamfaniabggHiLdGccqGGVaWlcqWGUbGBdaWgaaWcbaGaey4kaSIaem4Camhabeaaaaa@4776@. This relationship replaces R2 above.

If the CI for the directly standardized rates is based on a normal approximation, then from (2) we have that

c++n++<∑s=1Scisn+snisn++−z(α/2)∑s=1S(n+sn++)2cisnis2.     (10)
 MathType@MTEF@5@5@+=feaafiart1ev1aaatCvAUfKttLearuWrP9MDH5MBPbIqV92AaeXatLxBI9gBaebbnrfifHhDYfgasaacH8akY=wiFfYdH8Gipec8Eeeu0xXdbba9frFj0=OqFfea0dXdd9vqai=hGuQ8kuc9pgc9s8qqaq=dirpe0xb9q8qiLsFr0=vr0=vr0dc8meaabaqaciaacaGaaeqabaqabeGadaaakeaadaWcaaqaaiabdogaJnaaBaaaleaacqGHRaWkcqGHRaWkaeqaaaGcbaGaemOBa42aaSbaaSqaaiabgUcaRiabgUcaRaqabaaaaOGaeyipaWZaaabCaeaadaWcaaqaaiabdogaJnaaBaaaleaacqWGPbqAcqWGZbWCaeqaaOGaemOBa42aaSbaaSqaaiabgUcaRiabdohaZbqabaaakeaacqWGUbGBdaWgaaWcbaGaemyAaKMaem4Camhabeaakiabd6gaUnaaBaaaleaacqGHRaWkcqGHRaWkaeqaaaaaaeaacqWGZbWCcqGH9aqpcqaIXaqmaeaacqWGtbWua0GaeyyeIuoakiabgkHiTiabdQha6jabcIcaOGGaciab=f7aHjabc+caViabikdaYiabcMcaPmaakaaabaWaaabCaeaadaqadaqaamaalaaabaGaemOBa42aaSbaaSqaaiabgUcaRiabdohaZbqabaaakeaacqWGUbGBdaWgaaWcbaGaey4kaSIaey4kaScabeaaaaaakiaawIcacaGLPaaadaahaaWcbeqaaiabikdaYaaakmaalaaabaGaem4yam2aaSbaaSqaaiabdMgaPjabdohaZbqabaaakeaacqWGUbGBdaqhaaWcbaGaemyAaKMaem4CamhabaGaeGOmaidaaaaaaeaacqWGZbWCcqGH9aqpcqaIXaqmaeaacqWGtbWua0GaeyyeIuoaaSqabaGccqGGUaGlcaWLjaGaaCzcamaabmaabaGaeGymaeJaeGimaadacaGLOaGaayzkaaaaaa@7315@

is required for cell *i *to be significant. Similarly, if the gamma interval is used, *c*_++_/*n*_++ _<*v*_*i*_*x*_*iL*_/(2*y*_*i*_). These relations replace R3 for the respective CI method.

### Power calculations

We next consider the power of the CI and BN approaches using selected crude rates for different sample sizes and different true cell proportions of cases (*θ*). The power calculations can be obtained analytically for a hypothetical cell *i *and are compared when the observed cell cases are smaller or larger than the cluster size used for the BN approach.

Using the BN approach, cell *i *will be identified as a cell with a statistically elevated number of cases if P
 MathType@MTEF@5@5@+=feaafiart1ev1aaatCvAUfKttLearuWrP9MDH5MBPbIqV92AaeXatLxBI9gBamrtHrhAL1wy0L2yHvtyaeHbnfgDOvwBHrxAJfwnaebbnrfifHhDYfgasaacH8akY=wiFfYdH8Gipec8Eeeu0xXdbba9frFj0=OqFfea0dXdd9vqai=hGuQ8kuc9pgc9s8qqaq=dirpe0xb9q8qiLsFr0=vr0=vr0dc8meaabaqaciaacaGaaeqabaWaaeGaeaaakeGabaaUrGqaciab=bfaqbaa@385B@_*α*_(λ^
 MathType@MTEF@5@5@+=feaafiart1ev1aaatCvAUfKttLearuWrP9MDH5MBPbIqV92AaeXatLxBI9gBaebbnrfifHhDYfgasaacH8akY=wiFfYdH8Gipec8Eeeu0xXdbba9frFj0=OqFfea0dXdd9vqai=hGuQ8kuc9pgc9s8qqaq=dirpe0xb9q8qiLsFr0=vr0=vr0dc8meaabaqaciaacaGaaeqabaqabeGadaaakeaaiiGacuWF7oaBgaqcaaaa@2E77@_*i*:0_) + 1 ≤ *k*_*i *_≤ *c*_*i*+ _(using R1, R2, and (7)). The power of the test will be M1(θ)=1−∑u=0ki−1(θni+)uu!e−θni+
 MathType@MTEF@5@5@+=feaafiart1ev1aaatCvAUfKttLearuWrP9MDH5MBPbIqV92AaeXatLxBI9gBaebbnrfifHhDYfgasaacH8akY=wiFfYdH8Gipec8Eeeu0xXdbba9frFj0=OqFfea0dXdd9vqai=hGuQ8kuc9pgc9s8qqaq=dirpe0xb9q8qiLsFr0=vr0=vr0dc8meaabaqaciaacaGaaeqabaqabeGadaaakeaacqWGnbqtdaWgaaWcbaGaeGymaedabeaakiabcIcaOGGaciab=H7aXjabcMcaPiabg2da9iabigdaXiabgkHiTmaaqadabaWaaSaaaeaacqGGOaakcqWF4oqCcqWGUbGBdaWgaaWcbaGaemyAaKMaey4kaScabeaakiabcMcaPmaaCaaaleqabaGaemyDauhaaaGcbaGaemyDauNaeiyiaecaaaWcbaGaemyDauNaeyypa0JaeGimaadabaGaem4AaS2aaSbaaWqaaiabdMgaPbqabaWccqGHsislcqaIXaqma0GaeyyeIuoakiabdwgaLnaaCaaaleqabaGaeyOeI0Iae8hUdeNaemOBa42aaSbaaWqaaiabdMgaPjabgUcaRaqabaaaaaaa@52A8@ under the alternative hypothesis λ_*i*:0 _= *θ n*_*i*+_. Note that for the same value of *θ *if *k*_*i*1 _and *k*_*i*2 _are cluster sizes such that *k*_*i*1 _<*k*_*i*2 _and the null hypothesis is rejected for each of these cluster sizes, then then the smaller cluster size will have greater power (*M*_1_(*θ*, *k*_*i*2 _<*M*_1_(*θ*, *k*_*i*1_)).

The power function for the CI approach also requires values for the observed proportion of cases in the cell. The CI method compares the overall proportion of cases, *c*_++_/*n*_++_, with the observed cell proportion, *c*_*i*+_/*n*_*i*+_. The latter is used to construct the approximate confidence interval and the lower limit of the confidence interval becomes part of the power calculation for the one-sample test of proportion. The power of the test is

M2(θ)=1−Φ(c++n+++z(α/2)ci+ni+2(1−ci+ni+)−θθ(1−θ)/ni+)     (11)
 MathType@MTEF@5@5@+=feaafiart1ev1aaatCvAUfKttLearuWrP9MDH5MBPbIqV92AaeXatLxBI9gBaebbnrfifHhDYfgasaacH8akY=wiFfYdH8Gipec8Eeeu0xXdbba9frFj0=OqFfea0dXdd9vqai=hGuQ8kuc9pgc9s8qqaq=dirpe0xb9q8qiLsFr0=vr0=vr0dc8meaabaqaciaacaGaaeqabaqabeGadaaakeaacqWGnbqtdaWgaaWcbaGaeGOmaidabeaakiabcIcaOGGaciab=H7aXjabcMcaPiabg2da9iabigdaXiabgkHiTiabfA6agnaabmaabaWaaSaaaeaadaWcaaqaaiabdogaJnaaBaaaleaacqGHRaWkcqGHRaWkaeqaaaGcbaGaemOBa42aaSbaaSqaaiabgUcaRiabgUcaRaqabaaaaOGaey4kaSIaemOEaONaeiikaGIae8xSdeMaei4la8IaeGOmaiJaeiykaKYaaOaaaeaadaWcaaqaaiabdogaJnaaBaaaleaacqWGPbqAcqGHRaWkaeqaaaGcbaGaemOBa42aa0baaSqaaiabdMgaPjabgUcaRaqaaiabikdaYaaaaaGcdaqadaqaaiabigdaXiabgkHiTmaalaaabaGaem4yam2aaSbaaSqaaiabdMgaPjabgUcaRaqabaaakeaacqWGUbGBdaWgaaWcbaGaemyAaKMaey4kaScabeaaaaaakiaawIcacaGLPaaacqGHsislcqWF4oqCaSqabaaakeaadaGcaaqaaiab=H7aXjabcIcaOiabigdaXiabgkHiTiab=H7aXjabcMcaPiabc+caViabd6gaUnaaBaaaleaacqWGPbqAcqGHRaWkaeqaaaqabaaaaaGccaGLOaGaayzkaaGaaCzcaiaaxMaadaqadaqaaiabigdaXiabigdaXaGaayjkaiaawMcaaaaa@6D73@

for alternative cell proportion *θ*, where Φ is the cumulative distribution function of the standard normal distribution.

### Self-inflicted injury data

We use a dataset on self-inflicted injuries (SIIs) requiring medical attention from emergency departments (EDs) in the Canadian province of Alberta during the 1998/9 fiscal year. The dataset was extracted from the Ambulatory Care Classification System, which recorded all episodes of ambulatory care provided in Alberta hospitals such as ED presentations. Alberta was divided into 17 Regional Health Authorities (RHAs) that we use as cells (*I *= 17). The SII presentations used in the analysis were based on ICD9-CM [13] codes E950 to E959, "Suicide and Self-inflicted Poisoning" and "Suicide and Self-Inflicted Injury". A case was defined as an individual who presented to an Alberta ED at least once during the study period with a self-inflicted injury. The cell population sizes, cases, and distances between pairs of RHAs were provided by Alberta Health and Wellness, the provincial health authority. The distance between two RHAs was determined as the Euclidean distance between the centroids of the RHAs. We restrict our analysis to the pediatric population (< 18 years).

## Results and discussion

### Self-inflicted injury data

The total population and cases were 785,079 and 827, respectively, providing an overall provincial rate of 105.3 cases per 100,000 population. The RHAs had population sizes that were very inhomogeneous, ranging from 6,195 (RHA 14) to 232,460 (RHA 4). The number of cases ranged from 3 to 227 and also varied greatly from RHA to RHA. Table 1 displays the results for crude rates and their associated confidence intervals. The components of R3 are also provided. Cells 6 and 9 have 95% CIs that lie above the overall provincial rate and would be classified as areas of high rates. Note that these cells also satisfy the R3 criterion λ^
 MathType@MTEF@5@5@+=feaafiart1ev1aaatCvAUfKttLearuWrP9MDH5MBPbIqV92AaeXatLxBI9gBaebbnrfifHhDYfgasaacH8akY=wiFfYdH8Gipec8Eeeu0xXdbba9frFj0=OqFfea0dXdd9vqai=hGuQ8kuc9pgc9s8qqaq=dirpe0xb9q8qiLsFr0=vr0=vr0dc8meaabaqaciaacaGaaeqabaqabeGadaaakeaaiiGacuWF7oaBgaqcaaaa@2E77@_*i*:0 _<*c*_*i*+ _- *z*(*α*/2) ci+(ni+−ci+)/ni+
 MathType@MTEF@5@5@+=feaafiart1ev1aaatCvAUfKttLearuWrP9MDH5MBPbIqV92AaeXatLxBI9gBaebbnrfifHhDYfgasaacH8akY=wiFfYdH8Gipec8Eeeu0xXdbba9frFj0=OqFfea0dXdd9vqai=hGuQ8kuc9pgc9s8qqaq=dirpe0xb9q8qiLsFr0=vr0=vr0dc8meaabaqaciaacaGaaeqabaqabeGadaaakeaadaGcaaqaaiabdogaJnaaBaaaleaacqWGPbqAcqGHRaWkaeqaaOGaeiikaGIaemOBa42aaSbaaSqaaiabdMgaPjabgUcaRaqabaGccqGHsislcqWGJbWydaWgaaWcbaGaemyAaKMaey4kaScabeaakiabcMcaPiabc+caViabd6gaUnaaBaaaleaacqWGPbqAcqGHRaWkaeqaaaqabaaaaa@3F6B@. The other cells either have approximate 95% CIs that contain the overall provincial rate or are below.

**Table 1:** Population, cases, expected cases (λ^
 MathType@MTEF@5@5@+=feaafiart1ev1aaatCvAUfKttLearuWrP9MDH5MBPbIqV92AaeXatLxBI9gBaebbnrfifHhDYfgasaacH8akY=wiFfYdH8Gipec8Eeeu0xXdbba9frFj0=OqFfea0dXdd9vqai=hGuQ8kuc9pgc9s8qqaq=dirpe0xb9q8qiLsFr0=vr0=vr0dc8meaabaqaciaacaGaaeqabaqabeGadaaakeaaiiGacuWF7oaBgaqcaaaa@2E77@_*i*:0_), R3 value (*c*_*i*+ _- *z*(*α*/2)ci+(ni+−ci+)/ni+
 MathType@MTEF@5@5@+=feaafiart1ev1aaatCvAUfKttLearuWrP9MDH5MBPbIqV92AaeXatLxBI9gBaebbnrfifHhDYfgasaacH8akY=wiFfYdH8Gipec8Eeeu0xXdbba9frFj0=OqFfea0dXdd9vqai=hGuQ8kuc9pgc9s8qqaq=dirpe0xb9q8qiLsFr0=vr0=vr0dc8meaabaqaciaacaGaaeqabaqabeGadaaakeaadaGcaaqaaiabdogaJnaaBaaaleaacqWGPbqAcqGHRaWkaeqaaOGaeiikaGIaemOBa42aaSbaaSqaaiabdMgaPjabgUcaRaqabaGccqGHsislcqWGJbWydaWgaaWcbaGaemyAaKMaey4kaScabeaakiabcMcaPiabc+caViabd6gaUnaaBaaaleaacqWGPbqAcqGHRaWkaeqaaaqabaaaaa@3F6B@, and approximate 95% confidence intervals (CIs) for each RHA.

*i*	*c*_*i*+_	*n*_*i*+_	λ^ MathType@MTEF@5@5@+=feaafiart1ev1aaatCvAUfKttLearuWrP9MDH5MBPbIqV92AaeXatLxBI9gBaebbnrfifHhDYfgasaacH8akY=wiFfYdH8Gipec8Eeeu0xXdbba9frFj0=OqFfea0dXdd9vqai=hGuQ8kuc9pgc9s8qqaq=dirpe0xb9q8qiLsFr0=vr0=vr0dc8meaabaqaciaacaGaaeqabaqabeGadaaakeaaiiGacuWF7oaBgaqcaaaa@2E77@_*i*:0_	R3 value	95% CI
1	53	42,645	44.922	38.740	(0.0009,0.0016)
2	31	24,583	25.896	20.094	(0.0008,0.0017)
3	12	19,381	20.416	5.212	(0.0003,0.0010)
4	221	232,460	244.873	191.876	(0.0008,0.0011)
5	14	15,606	16.440	6.670	(0.0004,0.0014)
6	82	54,819	57.746	64.265	(0.0012,0.0018)*
7	24	28,694	30.226	14.402	(0.0005,0.0012)
8	25	26,740	28.168	15.205	(0.0006,0.0013)
9	22	11,436	12.047	12.816	(0.0011,0.0027)*
10	227	206,226	217.238	197.486	(0.0010,0.0012)
11	17	24,806	26.131	8.921	(0.0004,0.0010)
12	34	33,357	35.138	22.577	(0.0007,0.0014)
13	19	27,385	28.847	10.460	(0.0004,0.0010)
14	9	6,195	6.526	3.124	(0.0005,0.0024)
15	17	9,501	10.008	8.926	(0.0009,0.0026)
16	17	12,599	13.272	8.924	(0.0007,0.0020)
17	3	8,646	9.108	-0.394	(0.0000,0.0007)

Total	827	785,079			

The overall provincial rate is 105.3 cases per 100,000 population. An asterisk (*) denotes RHAs with rates higher than the provincial rates at the 5% level, unadjusted for multiple testing.

For the BN method, we chose cluster sizes to be 1.5 times the expected cases (1.5 *λ*_*i*:0_) for each cell, though other approaches can be used (e.g., [14]). Clusters centered at cells 6, 9, and 15 are all identified as areas with statistically higher rates than the overall provincial average (Table 2). Cells 9 and 15 are significant on their own (ℓ = 0), whereas the cluster centered at cell 6 is significant at the tested cluster size when combined with cell 9 (ℓ = 1). For this dataset, the significant areas all had ℓ = 0 or were combined with a cell that was significant on its own, but that need not be the case. It could be that two nearest neighbours do not have enough cases on their own to be clusters, but when combined together (ℓ = 1) the two are statistically significant for a cluster. This situation may happen more readily if the region is divided into a greater number of cells and the cells have smaller population sizes.

**Table 2:** Clustering results for each RHA (*i*). An asterisk (*) denotes significant clusters at the 5% level, unadjusted for multiple testing.

*i*	*k*_*i*_	ℓ	Nearest ℓ Neighbours	∑p=0ℓcip+ MathType@MTEF@5@5@+=feaafiart1ev1aaatCvAUfKttLearuWrP9MDH5MBPbIqV92AaeXatLxBI9gBaebbnrfifHhDYfgasaacH8akY=wiFfYdH8Gipec8Eeeu0xXdbba9frFj0=OqFfea0dXdd9vqai=hGuQ8kuc9pgc9s8qqaq=dirpe0xb9q8qiLsFr0=vr0=vr0dc8meaabaqaciaacaGaaeqabaqabeGadaaakeaadaaeWaqaaiabdogaJnaaBaaaleaacqWGPbqAdaWgaaadbaGaemiCaahabeaaliabgUcaRaqabaaabaGaemiCaaNaeyypa0JaeGimaadabaGaeS4eHWganiabggHiLdaaaa@388A@	λ^ MathType@MTEF@5@5@+=feaafiart1ev1aaatCvAUfKttLearuWrP9MDH5MBPbIqV92AaeXatLxBI9gBaebbnrfifHhDYfgasaacH8akY=wiFfYdH8Gipec8Eeeu0xXdbba9frFj0=OqFfea0dXdd9vqai=hGuQ8kuc9pgc9s8qqaq=dirpe0xb9q8qiLsFr0=vr0=vr0dc8meaabaqaciaacaGaaeqabaqabeGadaaakeaaiiGacuWF7oaBgaqcaaaa@2E77@_*i*:ℓ _	*p*-value
1	68	1	2	84	70.818	0.647
2	39	1	5	45	42.335	0.717
3	31	1	4	233	265.289	1.000
4	368	4	3, 5, 6, 1	382	384.396	0.805
5	25	1	4	235	261.312	1.000
6	87	1	9	104	69.793	0.026*
7	46	1	9	46	42.273	0.303
8	43	1	6	107	85.914	1.000
9	19	0		22	12.047	0.039*
10	326	3	9,7,6	355	317.257	0.319
11	40	1	10	244	243.368	1.000
12	53	1	10	261	252.376	1.000
13	44	2	14, 11	45	61.504	0.992
14	10	1	13	28	35.373	1.000
15	16	0		17	10.008	0.049*
16	20	1	17	20	22.379	0.721
17	14	1	16	20	22.379	0.977

For cell 6, the cluster size of 87 was larger than the observed number of cases (82) and condition R1 was not satisfied. The results of the two approaches for this cell are not directly comparable. Because cells 9 and 15 are significant when ℓ = 0, the analysis becomes directly comparable with the CI method in Table 1. The results for cell 9 agree since the conditions R1-R3 are all satisfied, but the results for cell 15 disagree. In particular for cell 15, conditions R1 and R2 are satisfied but R3 is not satisfied (10 is not less than 8). Consequently, in this example the BN method identifies one more area of statistically higher SII rates than the CI method and one area is identified only when combined with its nearest neighbour.

### Power comparison

To directly compare the power of each approach using equations *M*_1_(*θ*) and *M*_2_(*θ*), we have to specify the overall proportion for the region as well as the true proportion and observed number of cases for a particular cell. We considered two overall proportions (*μ *= 1/1000, 5/1000) to represent a rare disease and three cell population sizes (*n*_*i*+ _= 5000, 10000, 50000). We assumed that the true rate for the cell tested was 1.2, 1.5, or 2 times the overall rate (e.g., *θ *= 1.2 *μ*). To further add realism, the observed cell rate, *c*_*i*+_/*n*_*i*+_, was specified as 0.9, 1, or 1.1 times the true rate (e.g., 0.9 *μ*). This step was added since the observed number of cases are not necessarily the same as the expected number of cases. The cluster size used for comparison was 1.5 times the expected rate (i.e., 1.5 × *n*_*i*+ _× *μ*). The power calculations for these scenarios appear in Table 3. In general, the BN method appears to have larger power when the cluster size is relatively small and the observed number of cases is at least as large as the cluster size. Note the latter is a requirement for direct comparison of the approaches when ℓ = 0. The methods are quite similarly powered when the true cell rate is twice the overall rate and the overall rate and sample size are not too small.

**Table 3:** Power for the BN (*M*_1_(*θ*)) and Cl (*M*_2_(*θ*)) methods.

	*μ *= 1/1000					*μ *= 5/1000				
		
*n*_*i*+_	*θ*	*k*_*i*_	*c*_*i*+_/*n*_*i*+_	*M*_1_(*θ*)	M_2_(*θ*)	*θ*	*k*_*i*_	*c*_*i*+_/*n*_*i*+_	*M*_1_(*θ*)	M_2_(*θ*)
5000	0.0012	8	0.00108	*-*	0.073	0.0060	38	0.00540	*-*	0.172
5000	0.0012	8	0.00120	*-*	0.060	0.0060	38	0.00600	*-*	0.148
5000	0.0012	8	0.00132	*-*	0.050	0.0060	38	0.00660	*-*	0.127
5000	0.0015	8	0.00135	*-*	0.172	0.0075	38	0.00675	*-*	0.575
5000	0.0015	8	0.00150	*-*	0.148	0.0075	38	0.00750	*-*	0.535
5000	0.0015	8	0.00165	0.475	0.127	0.0075	38	0.00825	0.489	0.498
5000	0.0020	8	0.00180	0.780	0.391	0.0100	38	0.00900	0.966	0.955
5000	0.0020	8	0.00200	0.780	0.353	0.0100	38	0.01000	0.966	0.944
5000	0.0020	8	0.00220	0.780	0.318	0.0100	38	0.01100	0.966	0.933

10000	0.0012	15	0.00108	-	0.100	0.0060	75	0.00540	-	0.286
10000	0.0012	15	0.00120	-	0.083	0.0060	75	0.00600	-	0.253
10000	0.0012	15	0.00132	-	0.070	0.0060	75	0.00660	-	0.224
10000	0.0015	15	0.00135	-	0.285	0.0075	75	0.00675	-	0.850
10000	0.0015	15	0.00150	0.534	0.252	0.0075	75	0.00750	0.515	0.826
10000	0.0015	15	0.00165	0.534	0.223	0.0075	75	0.00825	0.515	0.800
10000	0.0020	15	0.00180	0.895	0.648	0.0100	75	0.00900	0.996	0.999
10000	0.0020	15	0.00200	0.895	0.610	0.0100	75	0.01000	0.996	0.999
10000	0.0020	15	0.00220	0.895	0.573	0.0100	75	0.01100	0.996	0.999

50000	0.0012	75	0.00108	-	0.285	0.0060	375	0.00540	-	0.850
50000	0.0012	75	0.00120	-	0.252	0.0060	375	0.00600	-	0.825
50000	0.0012	75	0.00132	-	0.223	0.0060	375	0.00660	-	0.800
50000	0.0015	75	0.00135	-	0.848	0.0075	375	0.00675	-	1.000
50000	0.0015	75	0.00150	0.515	0.824	0.0075	375	0.00750	0.507	1.000
50000	0.0015	75	0.00165	0.515	0.798	0.0075	375	0.00825	0.507	1.000
50000	0.0020	75	0.00180	0.996	0.999	0.0100	375	0.00900	1.000	1.000
50000	0.0020	75	0.00200	0.996	0.999	0.0100	375	0.01000	1.000	1.000
50000	0.0020	75	0.00220	0.996	0.998	0.0100	375	0.01100	1.000	1.000

The cell sample size (*n*_*i*+_), true cell proportion (*θ*), and observed cell proportion (*c*_*i*+_/*n*_*i*+_), when the overall proportion (*μ*) is 1/1000 or 5/1000 are reported. The dashes (-) indicate situations where the observed cases would not be at least as large as the cluster size and the cell may only be significant if combined with one or more neighbouring cells.

**Figure 1:**
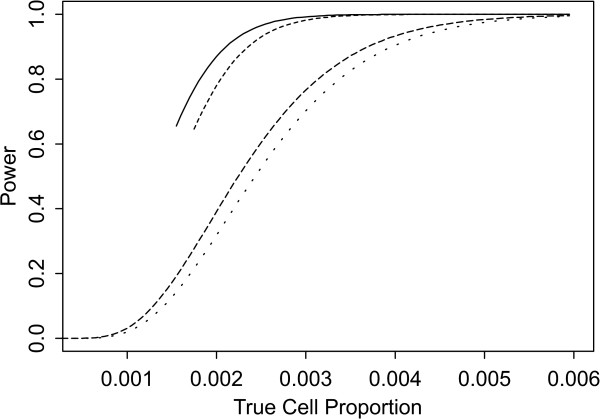
**Power for each method by true cell proportion (*θ*) when the overall proportion (*μ*) is 1/1000 and the cell population size (*n*_*i*+_) is 5000, without consideration of R1 and R2. **The solid and short dashed lines represent the BN method when the cluster sizes are 1.25 and 1.5 times the expected overall rate, respectively. The long dashed and dotted lines represent the CI method when the observed cell proportions (*c*_*i*+_/*n*_*i*+_) are 0.96*θ *and 1.1*θ*, respectively.

**Figure 2:**
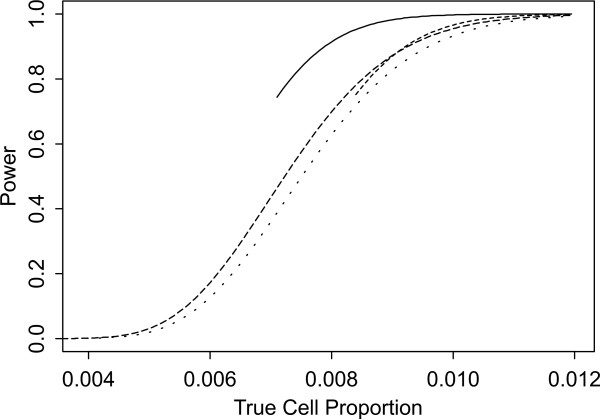
**Power for each method by true cell proportion (*θ*) when the overall proportion (*μ*) is 5/1000 and the cell population size (*n*_*i*+_) is 5000, without consideration of R1 and R2. **The solid and short dashed lines represent the BN method when the cluster sizes are 1.25 and 1.5 times the expected overall rate, respectively. The long dashed and dotted lines represent the CI method when the observed cell proportions (*c*_*i*+_/*n*_*i*+_) are 0.9*θ *and 1.1*θ*, respectively.

**Figure 3:**
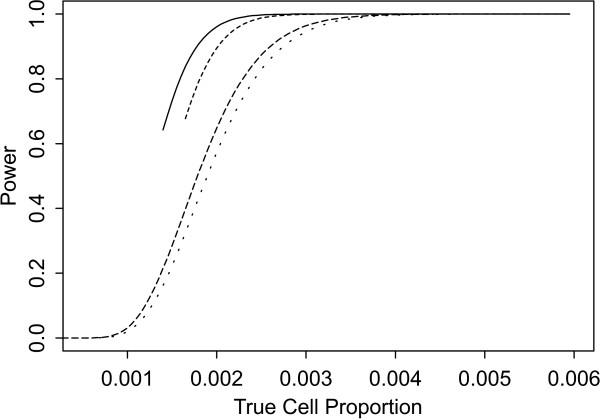
**Power for each method by true cell proportion (*θ*) when the overall proportion (*μ*) is 1/1000 and the cell population size (*n*_*i*+_) is 10000, without consideration of R1 and R2. **The solid and short dashed lines represent the BN method when the cluster sizes are 1.25 and 1.5 times the expected overall rate, respectively. The long dashed and dotted lines represent the CI method when the observed cell proportions (*c*_*i*+_/*n*_*i*+_) are 0.96*θ *and 1.1*θ*, respectively.

**Figure 4:**
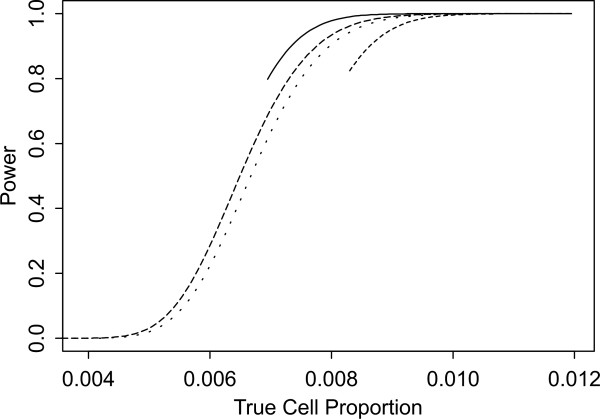
**Power for each method by true cell proportion (*θ*) when the overall proportion (*μ*) is 5/1000 and the cell population size (*n*_*i*+_) is 10000, without consideration of R1 and R2. **The solid and short dashed lines represent the BN method when the cluster sizes are 1.25 and 1.5 times the expected overall rate, respectively. The long dashed and dotted lines represent the CI method when the observed cell proportions (*c*_*i*+_/*n*_*i*+_) are 0.96*θ *and 1.1*θ*, respectively.

**Figure 5:**
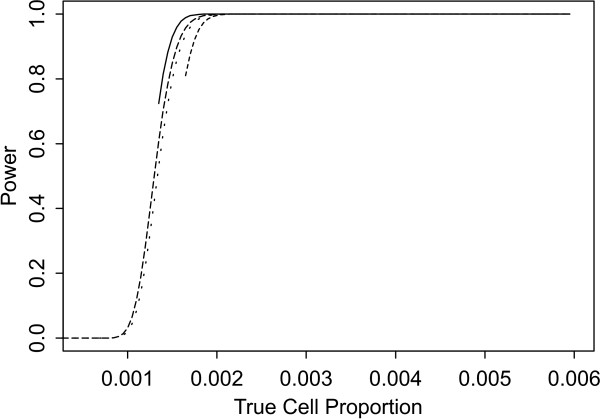
**Power for each method by true cell proportion (*θ*) when the overall proportion (*μ*) is 1/1000 and the cell population size (*n*_*i*+_) is 50000, without consideration of R1 and R2. **The solid and short dashed lines represent the BN method when the cluster sizes are 1.25 and 1.5 times the expected overall rate, respectively. The long dashed and dotted lines represent the CI method when the observed cell proportions (*c*_*i*+_/*n*_*i*+_) are 0.96*θ *and 1.1*θ*, respectively.

**Figure 6:**
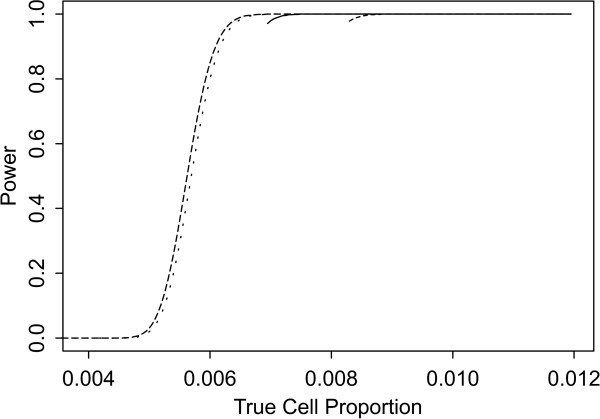
**Power for each method by true cell proportion (*θ*) when the overall proportion (*μ*) is 5/1000 and the cell population size (*n*_*i*+_) is 50000, without consideration of R1 and R2. **The solid and short dashed lines represent the BN method when the cluster sizes are 1.25 and 1.5 times the expected overall rate, respectively. The long dashed and dotted lines represent the CI method when the observed cell proportions (*c*_*i*+_/*n*_*i*+_) are 0.96*θ *and 1.1*θ*, respectively.

**Figure 7:**
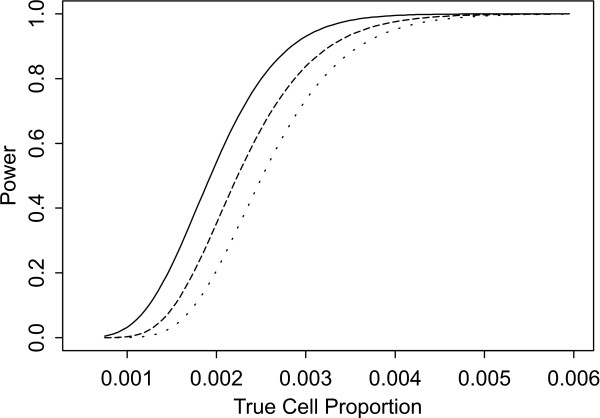
**Power for each method by true cell proportion (*θ*) when the overall proportion (*μ*) is 1/1000 and the cell population size (*n*_*i*+_) is 5000, when R1 and R2 are satisfied. **The solid lines represents the BN method when the cluster size *k*_*i *_= P
 MathType@MTEF@5@5@+=feaafiart1ev1aaatCvAUfKttLearuWrP9MDH5MBPbIqV92AaeXatLxBI9gBamrtHrhAL1wy0L2yHvtyaeHbnfgDOvwBHrxAJfwnaebbnrfifHhDYfgasaacH8akY=wiFfYdH8Gipec8Eeeu0xXdbba9frFj0=OqFfea0dXdd9vqai=hGuQ8kuc9pgc9s8qqaq=dirpe0xb9q8qiLsFr0=vr0=vr0dc8meaabaqaciaacaGaaeqabaWaaeGaeaaakeGabaaUrGqaciab=bfaqbaa@385B@_*α*_(λ^
 MathType@MTEF@5@5@+=feaafiart1ev1aaatCvAUfKttLearuWrP9MDH5MBPbIqV92AaeXatLxBI9gBaebbnrfifHhDYfgasaacH8akY=wiFfYdH8Gipec8Eeeu0xXdbba9frFj0=OqFfea0dXdd9vqai=hGuQ8kuc9pgc9s8qqaq=dirpe0xb9q8qiLsFr0=vr0=vr0dc8meaabaqaciaacaGaaeqabaqabeGadaaakeaaiiGacuWF7oaBgaqcaaaa@2E77@_*i*:0_) + 1. The dashed and dotted lines represent the CI method when the observed cell proportions (*c*_*i*+_/*n*_*i*+_) are *k*_*i*_/*n*_*i*+ _and *k*_*i*_/*n*_*i*+ _+ *c*_++_/*n*_++_, respectively.

**Figure 8:**
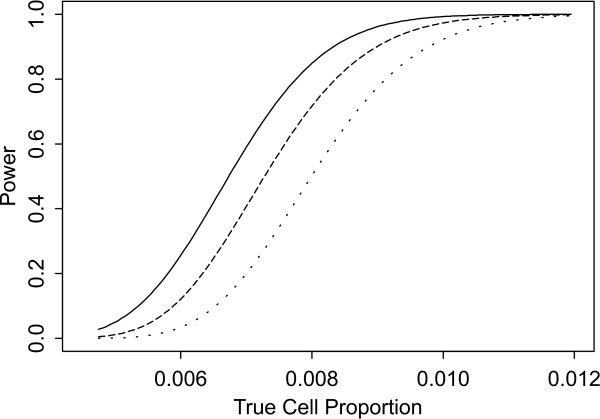
**Power for each method by true cell proportion (*θ*) when the overall proportion (*μ*) is 5/1000 and the cell population size (*n*_*i*+_) is 5000, when R1 and R2 are satisfied. **The solid lines represents the BN method when the cluster size *k*_*i *_= P
 MathType@MTEF@5@5@+=feaafiart1ev1aaatCvAUfKttLearuWrP9MDH5MBPbIqV92AaeXatLxBI9gBamrtHrhAL1wy0L2yHvtyaeHbnfgDOvwBHrxAJfwnaebbnrfifHhDYfgasaacH8akY=wiFfYdH8Gipec8Eeeu0xXdbba9frFj0=OqFfea0dXdd9vqai=hGuQ8kuc9pgc9s8qqaq=dirpe0xb9q8qiLsFr0=vr0=vr0dc8meaabaqaciaacaGaaeqabaWaaeGaeaaakeGabaaUrGqaciab=bfaqbaa@385B@_*α*_(λ^
 MathType@MTEF@5@5@+=feaafiart1ev1aaatCvAUfKttLearuWrP9MDH5MBPbIqV92AaeXatLxBI9gBaebbnrfifHhDYfgasaacH8akY=wiFfYdH8Gipec8Eeeu0xXdbba9frFj0=OqFfea0dXdd9vqai=hGuQ8kuc9pgc9s8qqaq=dirpe0xb9q8qiLsFr0=vr0=vr0dc8meaabaqaciaacaGaaeqabaqabeGadaaakeaaiiGacuWF7oaBgaqcaaaa@2E77@_*i*:0_) + 1. The dashed and dotted lines represent the CI method when the observed cell proportions (*c*_*i*+_/*n*_*i*+_) are *k*_*i*_/*n*_*i*+ _and *k*_*i*_/*n*_*i*+ _+ *c*_++_/*n*_++_, respectively.

**Figure 9:**
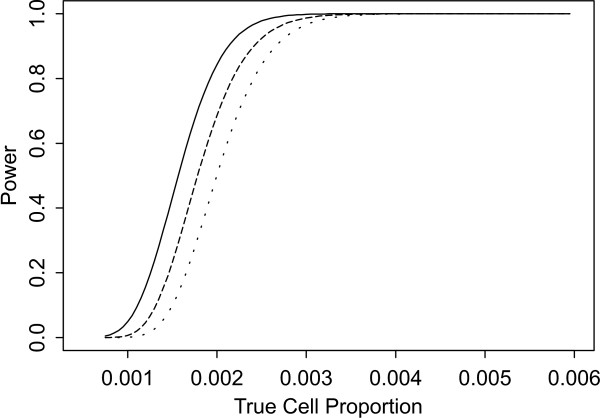
**Power for each method by true cell proportion (*θ*) when the overall proportion (*μ*) is 1/1000 and the cell population size (*n*_*i*+_) is 10000, when R1 and R2 are satisfied. **The solid lines represents the BN method when the cluster size *k*_*i *_= P
 MathType@MTEF@5@5@+=feaafiart1ev1aaatCvAUfKttLearuWrP9MDH5MBPbIqV92AaeXatLxBI9gBamrtHrhAL1wy0L2yHvtyaeHbnfgDOvwBHrxAJfwnaebbnrfifHhDYfgasaacH8akY=wiFfYdH8Gipec8Eeeu0xXdbba9frFj0=OqFfea0dXdd9vqai=hGuQ8kuc9pgc9s8qqaq=dirpe0xb9q8qiLsFr0=vr0=vr0dc8meaabaqaciaacaGaaeqabaWaaeGaeaaakeGabaaUrGqaciab=bfaqbaa@385B@_*α*_(λ^
 MathType@MTEF@5@5@+=feaafiart1ev1aaatCvAUfKttLearuWrP9MDH5MBPbIqV92AaeXatLxBI9gBaebbnrfifHhDYfgasaacH8akY=wiFfYdH8Gipec8Eeeu0xXdbba9frFj0=OqFfea0dXdd9vqai=hGuQ8kuc9pgc9s8qqaq=dirpe0xb9q8qiLsFr0=vr0=vr0dc8meaabaqaciaacaGaaeqabaqabeGadaaakeaaiiGacuWF7oaBgaqcaaaa@2E77@_*i*:0_) + 1. The dashed and dotted lines represent the CI method when the observed cell proportions (*c*_*i*+_/*n*_*i*+_) are *k*_*i*_/*n*_*i*+ _and *k*_*i*_/*n*_*i*+ _+ *c*_++_/*n*_++_, respectively.

**Figure 10:**
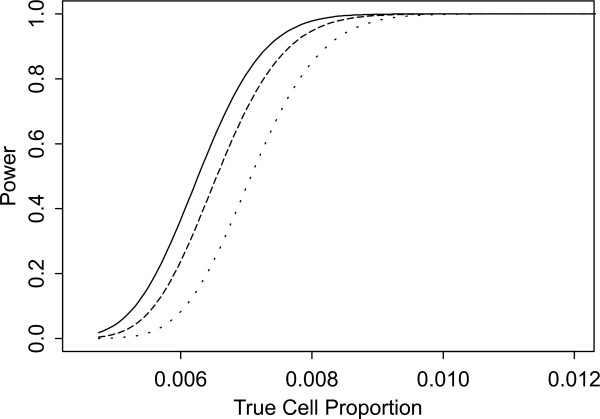
**Power for each method by true cell proportion (*θ*) when the overall proportion (*μ*) is 5/1000 and the cell population size (*n*_*i*+_) is 10000, when R1 and R2 are satisfied. **The solid lines represents the BN method when the cluster size *k*_*i *_= P
 MathType@MTEF@5@5@+=feaafiart1ev1aaatCvAUfKttLearuWrP9MDH5MBPbIqV92AaeXatLxBI9gBamrtHrhAL1wy0L2yHvtyaeHbnfgDOvwBHrxAJfwnaebbnrfifHhDYfgasaacH8akY=wiFfYdH8Gipec8Eeeu0xXdbba9frFj0=OqFfea0dXdd9vqai=hGuQ8kuc9pgc9s8qqaq=dirpe0xb9q8qiLsFr0=vr0=vr0dc8meaabaqaciaacaGaaeqabaWaaeGaeaaakeGabaaUrGqaciab=bfaqbaa@385B@_*α*_(λ^
 MathType@MTEF@5@5@+=feaafiart1ev1aaatCvAUfKttLearuWrP9MDH5MBPbIqV92AaeXatLxBI9gBaebbnrfifHhDYfgasaacH8akY=wiFfYdH8Gipec8Eeeu0xXdbba9frFj0=OqFfea0dXdd9vqai=hGuQ8kuc9pgc9s8qqaq=dirpe0xb9q8qiLsFr0=vr0=vr0dc8meaabaqaciaacaGaaeqabaqabeGadaaakeaaiiGacuWF7oaBgaqcaaaa@2E77@_*i*:0_) + 1. The dashed and dotted lines represent the CI method when the observed cell proportions (*c*_*i*+_/*n*_*i*+_) are *k*_*i*_/*n*_*i*+ _and *k*_*i*_/*n*_*i*+ _+ *c*_++_/*n*_++_, respectively.

**Figure 11:**
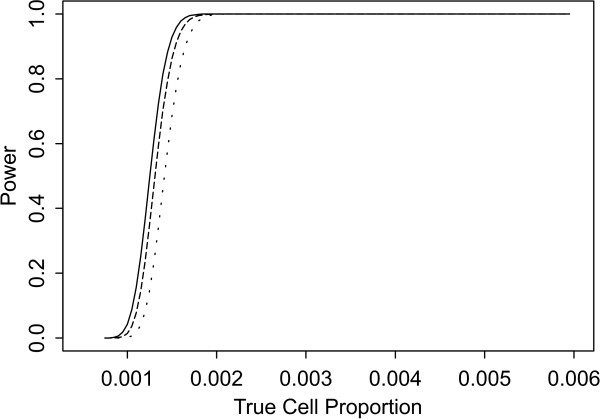
**Power for each method by true cell proportion (*θ*) when the overall proportion (*μ*) is 1/1000 and the cell population size (*n*_*i*+_) is 50000, when R1 and R2 are satisfied. **The solid lines represents the BN method when the cluster size *k*_*i *_= P
 MathType@MTEF@5@5@+=feaafiart1ev1aaatCvAUfKttLearuWrP9MDH5MBPbIqV92AaeXatLxBI9gBamrtHrhAL1wy0L2yHvtyaeHbnfgDOvwBHrxAJfwnaebbnrfifHhDYfgasaacH8akY=wiFfYdH8Gipec8Eeeu0xXdbba9frFj0=OqFfea0dXdd9vqai=hGuQ8kuc9pgc9s8qqaq=dirpe0xb9q8qiLsFr0=vr0=vr0dc8meaabaqaciaacaGaaeqabaWaaeGaeaaakeGabaaUrGqaciab=bfaqbaa@385B@_*α*_(λ^
 MathType@MTEF@5@5@+=feaafiart1ev1aaatCvAUfKttLearuWrP9MDH5MBPbIqV92AaeXatLxBI9gBaebbnrfifHhDYfgasaacH8akY=wiFfYdH8Gipec8Eeeu0xXdbba9frFj0=OqFfea0dXdd9vqai=hGuQ8kuc9pgc9s8qqaq=dirpe0xb9q8qiLsFr0=vr0=vr0dc8meaabaqaciaacaGaaeqabaqabeGadaaakeaaiiGacuWF7oaBgaqcaaaa@2E77@_*i*:0_) + 1. The dashed and dotted lines represent the CI method when the observed cell proportions (*c*_*i*+_/*n*_*i*+_) are *k*_*i*_/*n*_*i*+ _and *k*_*i*_/*n*_*i*+ _+ *c*_++_/*n*_++_, respectively.

**Figure 12:**
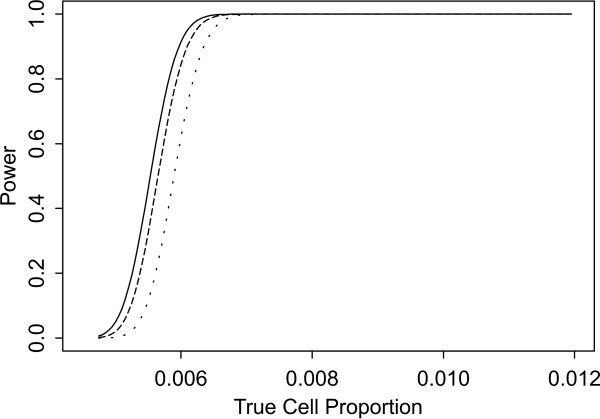
**Power for each method by true cell proportion (*θ*) when the overall proportion (*μ*) is 5/1000 and the cell population size (*n*_*i*+_) is 50000, when R1 and R2 are satisfied. **The solid lines represents the BN method when the cluster size *k*_*i *_= P
 MathType@MTEF@5@5@+=feaafiart1ev1aaatCvAUfKttLearuWrP9MDH5MBPbIqV92AaeXatLxBI9gBamrtHrhAL1wy0L2yHvtyaeHbnfgDOvwBHrxAJfwnaebbnrfifHhDYfgasaacH8akY=wiFfYdH8Gipec8Eeeu0xXdbba9frFj0=OqFfea0dXdd9vqai=hGuQ8kuc9pgc9s8qqaq=dirpe0xb9q8qiLsFr0=vr0=vr0dc8meaabaqaciaacaGaaeqabaWaaeGaeaaakeGabaaUrGqaciab=bfaqbaa@385B@_*α*_(λ^
 MathType@MTEF@5@5@+=feaafiart1ev1aaatCvAUfKttLearuWrP9MDH5MBPbIqV92AaeXatLxBI9gBaebbnrfifHhDYfgasaacH8akY=wiFfYdH8Gipec8Eeeu0xXdbba9frFj0=OqFfea0dXdd9vqai=hGuQ8kuc9pgc9s8qqaq=dirpe0xb9q8qiLsFr0=vr0=vr0dc8meaabaqaciaacaGaaeqabaqabeGadaaakeaaiiGacuWF7oaBgaqcaaaa@2E77@_*i*:0_) + 1. The dashed and dotted lines represent the CI method when the observed cell proportions (*c*_*i*+_/*n*_*i*+_) are *k*_*i*_/*n*_*i*+ _and *k*_*i*_/*n*_*i*+ _+ *c*_++_/*n*_++_, respectively.

Two sets of power curves are provided for selected scenarios in Figures 1, 2, 3, 4, 5, 6, 7, 8, 9, 10, 11, 12. The cell population sizes and overall proportions are the same as in Table 3. In Figures 1, 2, 3, 4, 5, 6, the cluster sizes for the BN method are chosen to represent observed cases at least as large as 1.25 and 1.5 times the expected rate (i.e., 1.25 × *n*_*i*+ _× *μ*). For the CI method, the observed cell proportion is taken as a factor times the true cell proportion, either 0.9 *θ *or 1.1 *θ *. We see that for the lower overall rate and smaller sample sizes the BN method has larger power than the CI method, provided the observed cases exceed the cluster size (i.e., R1 satisfied). Alternatively, if both R1 and R2 are satisfied, the power curves are provided in Figures 7, 8, 9, 10, 11, 12. To directly compare the two methods, the cluster size and observed cases are chosen so that R1 and R2 are satisfied, *k*_*i *_= P
 MathType@MTEF@5@5@+=feaafiart1ev1aaatCvAUfKttLearuWrP9MDH5MBPbIqV92AaeXatLxBI9gBamrtHrhAL1wy0L2yHvtyaeHbnfgDOvwBHrxAJfwnaebbnrfifHhDYfgasaacH8akY=wiFfYdH8Gipec8Eeeu0xXdbba9frFj0=OqFfea0dXdd9vqai=hGuQ8kuc9pgc9s8qqaq=dirpe0xb9q8qiLsFr0=vr0=vr0dc8meaabaqaciaacaGaaeqabaWaaeGaeaaakeGabaaUrGqaciab=bfaqbaa@385B@_*α*_(λ^
 MathType@MTEF@5@5@+=feaafiart1ev1aaatCvAUfKttLearuWrP9MDH5MBPbIqV92AaeXatLxBI9gBaebbnrfifHhDYfgasaacH8akY=wiFfYdH8Gipec8Eeeu0xXdbba9frFj0=OqFfea0dXdd9vqai=hGuQ8kuc9pgc9s8qqaq=dirpe0xb9q8qiLsFr0=vr0=vr0dc8meaabaqaciaacaGaaeqabaqabeGadaaakeaaiiGacuWF7oaBgaqcaaaa@2E77@_*i*:0_) + 1 and *k*_*i *_≤ *c*_*i*+_. In addition, the observed cell proportions are chosen to be *k*_*i*_/*n*_*i*+ _and *k*_*i*_/*n*_*i*+ _+ *c*_++_/*n*_++_. In the latter situation, R3 is satisfied as well. The BN method has greater power than the CI method in these situations.

## Conclusion

Health authorities often monitor the distribution of disease in a geographic region consisting of several sub-regions called cells. Cells with higher than expected disease cases may be the result of an environmental cause. In order to determine the cause for elevated cases, thorough epidemiological investigations can commence. Surveillance methods allow cells to be targeted for these investigations. Two common approaches emerge: disease rates and their associated confidence intervals are compared with the overall rate or statistical disease cluster detection methods identify areas of excess disease. The disease rates and confidence intervals are easily calculated using standard statistical software, but are not designed to detect clusters and ignore the spatial relationship among the cells, a serious drawback. The cluster detection methods often need the user to create specialized computer programs or use more sophisticated statistical software (e.g., R [15] is free software with functions for the BN method). Additionally, the cluster detection methods require additional information related to the spatial relationship among areas to enable the combination of cells. Comparison of the approaches is an important aspect in order to determine when the cluster detection method yields different results than merely rate and confidence interval calculations. These approaches are only comparable when an individual cell is identified as a cluster.

We focused this paper on CI methods for crude and directly standardized rates and the non-focused test developed by Besag and Newell [1] to detect clusters. The latter was chosen because it requires aggregate information, a simplified spatial relationship in the form of ordered nearest neighbours, and combines populations of intact areas. This approach is most comparable with the CI methods for rates and thus, a natural question arises of when these methods would identify the same areas as having statistically higher rates. We showed that there are three simple conditions that must be satisfied in order for the same areas to be identified as having higher crude rates than the overall regional rate. Two of the conditions need adaptations for the analyses based on directly standardized rates. These conditions are easily calculated since they are based on quantities already calculated for the CI methods. We illustrated the methods on an emergency department dataset on individuals seeking medical treatment for self-inflicted injuries.

A key aspect to the conditions for agreement is the cluster size. If the cluster size is larger than the number of cases in the cell, the BN method requires cells to be combined and the combination means that the results will be different from the CI method. In this situation, the BN method is preferable and the extra effort is required. Conversely, if the cluster size is less than the observed number of cases in a cell, then the conditions provide the foundation for when the BN and CI methods agree. While it is advised to choose the cluster size before knowing the actual cases observed, once chosen the actual cases could be compared and the conditions examined before proceeding with the BN method. The choice of cluster size is an important issue in statistical methods for disease cluster detection and our comparison suggests that the cluster size needs to be chosen to reflect an *a priori *medically meaningful size and often be larger than the number of actual cases within a cell. The power comparisons further illustrated this important aspect.

The choice of method will also depend on the incidence or prevalence of the disease examined and the population sizes of the cell. If the disease is rare and the population of the cell is small, the CI approach would be based on rates that may not be stable and the approach will have low power. In this situation, the BN approach would be more appropriate because the combination of neighbouring cells serves to increase the sample size, resulting in more stable rates and higher power. Conversely, if the cell population sizes are large, the BN method may not require any combination of cells and would be directly comparable to the CI method. In the latter instance, the rate estimates would be more stable but sub-areas within the cell that have higher rates cannot be identified.

## Competing interests

The author(s) declare that they have no competing interests.

## Authors' contributions

RJR conceived the idea, drafted the manuscript, provided the mathematical criteria, developed the computer programs, and obtained the results.
